# Decoding the Impact of a Bacterial Strain of *Micrococcus luteus* on *Arabidopsis* Growth and Stress Tolerance

**DOI:** 10.3390/microorganisms12112283

**Published:** 2024-11-10

**Authors:** Yu-Cheng Chang, Pin-Hsueh Lee, Chao-Liang Hsu, Wen-Der Wang, Yueh-Long Chang, Huey-wen Chuang

**Affiliations:** Department of Agricultural Biotechnology, National Chiayi University, Chiayi 600355, Taiwan; s1120111@mail.ncyu.edu.tw (Y.-C.C.); lee5313@purdue.edu (P.-H.L.); jason81334@gmail.com (C.-L.H.); wangw4@mail.ncyu.edu.tw (W.-D.W.); yuehlong@mail.ncyu.edu.tw (Y.-L.C.)

**Keywords:** microbial IAA, microbial carotenoids, auxin signal, ABA signal

## Abstract

Microbes produce various bioactive metabolites that can influence plant growth and stress tolerance. In this study, a plant growth-promoting rhizobacterium (PGPR), strain S14, was identified as *Micrococcus luteus* (designated as MlS14) using de novo whole-genome assembly. The MlS14 genome revealed major gene clusters for the synthesis of indole-3-acetic acid (IAA), terpenoids, and carotenoids. MlS14 produced significant amounts of IAA, and its volatile organic compounds (VOCs), specifically terpenoids, exhibited antifungal activity, suppressing the growth of pathogenic fungi. The presence of yellow pigment in the bacterial colony indicated carotenoid production. Treatment with MlS14 activated the expression of *β-glucuronidase* (*GUS*) driven by a promoter containing auxin-responsive elements. The application of MlS14 reshaped the root architecture of *Arabidopsis* seedlings, causing shorter primary roots, increased lateral root growth, and longer, denser root hairs; these characteristics are typically controlled by elevated exogenous IAA levels. MlS14 positively regulated seedling growth by enhancing photosynthesis, activating antioxidant enzymes, and promoting the production of secondary metabolites with reactive oxygen species (ROS) scavenging activity. Pretreatment with MlS14 reduced H_2_O_2_ and malondialdehyde (MDA) levels in seedlings under drought and heat stress, resulting in greater fresh weight during the post-stress period. Additionally, exposure to MlS14 stabilized chlorophyll content and growth rate in seedlings under salt stress. MlS14 transcriptionally upregulated genes involved in antioxidant defense and photosynthesis. Furthermore, genes linked to various hormone signaling pathways, such as abscisic acid (ABA), auxin, jasmonic acid (JA), and salicylic acid (SA), displayed increased expression levels, with those involved in ABA synthesis, using carotenoids as precursors, being the most highly induced. Furthermore, MlS14 treatment increased the expression of several transcription factors associated with stress responses, with *DREB2A* showing the highest level of induction. In conclusion, MlS14 played significant roles in promoting plant growth and stress tolerance. Metabolites such as IAA and carotenoids may function as positive regulators of plant metabolism and hormone signaling pathways essential for growth and adaptation to abiotic stress.

## 1. Introduction

Rhizobacteria from different ecological niches exhibit diverse interactions with their host plants, driven by the wide array of microbial metabolites that define these distinctive plant–microbe relationships [1]. Indole-3-acetic acid (IAA), the most common auxin, is produced by various microorganisms [2]. Auxin, a plant hormone, is essential for root growth and development by regulating root gravitropism, facilitating lateral root formation, and promoting root hair development [3]. Microorganisms isolated from the rhizosphere of different crops can synthesize IAA as a secondary metabolite, promoting the growth of lateral roots and root hairs, which are essential for nutrient uptake [4]. Siderophores are small molecules synthesized by bacteria to capture iron from the environment. This process can enhance iron absorption in plants colonized by these microorganisms. Since iron is essential for all living organisms, siderophores sequester iron, reducing its availability to plant pathogens and thereby indirectly promoting plant growth and health [5].

Volatile organic compounds (VOCs) produced by microorganisms function as signaling molecules, facilitating communication among different microbial species. Additionally, these microbial VOCs can inhibit or eliminate competing fungi or bacteria [6]. Moreover, VOCs emitted by rhizobacteria have the ability to modify root architecture in host plants, thereby enhancing plant growth [7]. These VOCs can also enhance plant defense responses against pathogens by triggering phytohormone-dependent signaling pathways [8]. Certain VOCs, such as acetoin and 2,3-butanediol, can promote plant growth by stimulating root development, enhancing nutrient uptake, and triggering systemic resistance against pathogens [9]. Microorganisms also synthesize various types of terpenoid VOCs that exhibit antioxidant and antimicrobial activities [10].

Microorganisms produce various types of pigment compounds that are crucial for their survival, protection, and interactions within their ecosystems. For example, carotenoids are yellow, orange, or red pigments that protect microorganisms against oxidative damage and UV radiation [11]. In plant cells, carotenoid pigments have antioxidant properties that help reduce oxidative stress and improve photosynthetic efficiency [12]. Spermidine is a polyamine that performs several essential functions in microorganisms, including facilitating biofilm formation, which is crucial for surface attachment, and providing protection against environmental stressors such as oxidative stress [13]. Microbial spermidine can function as a stress alleviator in colonized plants. Spermidine produced by *Bacillus megaterium* increases plant abscisic acid (ABA) levels, promoting plant growth and enhancing drought stress tolerance [14]. Furthermore, spermidine secreted by *B. subtilis* strain OKB105 reduces plant ethylene levels, consequently enhancing plant growth [15]. Ethylene is a gaseous plant hormone that plays a significant role in stress-related responses, including growth inhibition, senescence, and tissue damage [16].

In natural environments, plants are subjected to various biotic and abiotic stresses. A common response to these stressors is the accumulation of reactive oxygen species (ROS), which can negatively impact plant growth and development [17]. Various plant hormones orchestrate adaptive physiological responses that enable plants to withstand the adverse effects of environmental stress. Among these, ABA is a key plant hormone that regulates adaptive responses to various abiotic stress conditions [18]. It enhances stress tolerance by controlling stomatal closure to reduce water loss during osmotic stress, modifying root architecture to improve water and nutrient uptake, and activating antioxidant systems to counteract oxidative stress induced by environmental challenges [19,20,21]. Salicylic acid (SA) and jasmonic acid (JA) are crucial signaling molecules in plant defense mechanisms, with SA primarily involved in systemic acquired resistance (SAR) and JA playing a key role in induced systemic resistance (ISR) [22]. Additional roles of SA and JA in regulating abiotic stress tolerance have been identified. Both SA and JA can enhance antioxidant activity to manage oxidative stress under stressful environmental conditions [23,24]. Accumulating evidence supports that PGPR are highly effective at enhancing plant tolerance to abiotic stress. The rhizobacterial strain *B. mycoides* enhances tolerance to drought and heat stress and activates JA and SA signaling pathways in *Arabidopsis* [25]. The *B. licheniformis* strain CH102, which produces significant antifungal VOCs, improves plant growth vigor under dehydration and high-temperature conditions. The application of this strain activated genes associated with the JA and ABA signaling pathways [26]. Additionally, a spermidine-producing *B. megaterium* strain activates ABA signaling to improve plant tolerance to water deficit [14].

*Micrococcus* bacterial strains isolated from diverse environments exhibit the ability to break down xenobiotic compounds and produce compounds with antioxidant and antimicrobial properties [11,27]. The *Micrococcus* genus has the ability to survive in extreme environments. *M. luteus* SA211 has been identified as capable of tolerating high concentrations of LiCl [28]. The *M. luteus* strain AS2 is resistant to heavy metals [27]. However, the bioactive metabolites produced by *M. luteus* that promote plant growth and enhance stress tolerance remain unclear. In this study, MlS14 was identified as a new rhizobacterial strain of *M. luteus*. Using de novo whole-genome assembly methods, three major gene groups associated with the synthesis of metabolites, including IAA, terpenoids, and carotenoids, were identified in the MlS14 genome. The plant growth-promoting functions of MlS14 were confirmed in *Arabidopsis* seedlings, including improvements in root development, enhanced photosynthetic efficiency, and the activation of antioxidant defenses. *Arabidopsis*, a well-established model organism, offers in-depth insights into the molecular mechanisms that respond to abiotic stress [29]. Our results show that MlS14 treatment increased tolerance to abiotic stresses such as drought, heat, and salinity. Additionally, MlS14 triggered the expression of genes involved in various hormone signaling pathways, including ABA, auxin, JA, and SA.

## 2. Materials and Methods

### 2.1. Identification of the Bacterial Strain

#### 2.1.1. Analysis of 16S rDNA Sequence

The bacterial strain S14 was isolated from compost prepared as a plant growth substrate. The bacterial genomic DNA was isolated by following the procedure described by Griffiths et al. (2000) [30]. The PCR fragment using primers targeting the 16S rDNA sequence was analyzed using a 3730 DNA Analyzer (Applied Biosystems^®^; Foster City, CA, USA). The forward and reverse PCR primer sequences were 5′AGAGTTTGATCCTGGCTCAG3′ and 5′ACGGTTACCTTGTTACGACTT3′, respectively. The 16S rDNA sequences were examined using the Basic Local Alignment Search Tool (BLAST) available in the NCBI database (https://blast.ncbi.nlm.nih.gov/Blast.cgi, accessed on 1 September 2023). The genomic DNA of strain S14 was fragmented and used to prepare the whole genome sequence assembly of strain S14, following the procedures described in Chang et al. (2024) [31].

#### 2.1.2. De Novo Whole Genome Assembly

Bacterial genomic DNA was fragmented, and fragments exceeding 400 bp were purified for the construction of a genomic DNA library using the Celero DNA-Seq Library Preparation Kit (Tecan Genomics, CA, USA). Paired-end DNA sequencing was performed using the Illumina MiSeq system (Illumina, Inc., San Diego, CA, USA). The resulting DNA sequences were analyzed according to the procedures described by Tsai et al. (2023) [32].

### 2.2. Analysis of Bioactivity of Microbial Metabolites

#### 2.2.1. IAA Production

The isolated strain was cultured on nutrient agar (NA) and in nutrient broth (NB) medium for 3 days to assess its bacterial morphology. To measure the IAA concentration, 1 mL of the supernatant from bacterial cultures grown in LB broth with tryptophan (2 mg/mL) for 24, 48, and 72 h was combined with 2 mL of Salkowski reagent [33] and left to react at room temperature for 30 min. The absorbance at 530 nm was measured, and the IAA concentration was determined by comparison with a standard curve.

#### 2.2.2. Antifungal Activity

To detect the antifungal activity of volatile metabolites produced by isolated bacterial strain, methods outlined by Tsai et al. (2023) were adopted [32]. The tested bacterial strain and the targeted fungal pathogens, including *Fusarium oxysporum* f. sp. *cubense* tropical race 4 (*Foc* TR4) and *F. solani*, were cultured separately on PDA medium. The fungal plate was inverted and positioned on top of the bacterial plate, then sealed with parafilm. These co-cultures of the tested bacterial strain and targeted fungal pathogens were incubated at 28 °C for 4 days. The inhibition rate (I) was calculated using the formula I = (1 − T/C) × 100, where C and T represent the mycelium diameter of fungal pathogens co-cultured with H_2_O and the tested bacterial strain, respectively. The mean and standard error of the mycelium inhibition rate were determined based on the results from three replicates.

#### 2.2.3. Salt Stress Resistance and Phosphate Solubilizing Activity

To analyze salt stress resistance, the bacterial strain was cultured in LB medium supplemented with NaCl to achieve final concentrations of 1%, 2%, 4%, 6%, 8%, and 10%. The absorbance of the bacterial culture at 600 nm was measured after overnight incubation. To analyze phosphate solubilizing activity, Pilovskaya’s (PVK) agar medium was prepared according to the method described by Chang et al. (2023) [34]. A single colony was inoculated at the center of the Pilovskaya’s (PVK) agar plate and incubated at 28 °C for 14 days. The formation of a clear zone around the colony indicates phosphate solubilizing activity. To quantify phosphate solubilizing activity, supernatants obtained from bacterial cultures grown in PVK broth for 3 days were mixed with Vanadate–Molybdate reagent, and the absorbance was measured at 470 nm after 1 h of reaction. Phosphate solubilizing activity was then determined using a standard curve.

#### 2.2.4. Nitrogen Fixation Activity

Qualitative analysis of nitrogen fixation activity was conducted by inoculating a single bacterial colony into Jensen’s medium containing 2% sucrose, 0.1% _K2_HPO_4_, 0.05% MgSO_4_, 0.5% NaCl, 0.01% FeSO_4_, 0.0005% Na_2_MoO_4_·2H_2_O, 0.2% CaCO_3_, and 0.04% bromothymol blue, and incubating it at 28 °C for 3 days. The nitrogen-fixing ability of the strain was evaluated based on the color change of the medium. This nitrogen fixation activity was quantified by adding the bacterial culture to Jensen’s medium and culturing for an additional 3 days. Supernatants from the bacterial cultures were collected, and the absorbance at 630 nm was measured. Percentage of biological nitrogen fixation (BNF) was calculated using the formula (OD_reference_ − OD_sample_)/OD_reference_ × 100, as described by Sharma and Saharan (2017) [35].

### 2.3. Plant Growth Promotion Analysis

#### 2.3.1. Histochemical Staining of β-Glucuronidase (GUS)

Four-day-old seedlings of *Arabidopsis thaliana* containing the BA3::GUS construct were co-cultured with the tested bacterial strain for six days at 23 °C under a 16 h light condition. The histochemical analysis of GUS activity was conducted by following the methods described by Jefferson et al. (1987) [36]. The seedlings were then soaked in a staining solution containing 50 mM NaHPO_4_ (pH 7.0), 1 mM 5-bromo-4-chloro-3-indolyl-β-D-glucuronide (X-Gluc), 0.5 mM K_3_Fe(CN)_6_, 0.5 mM K₄Fe(CN)_6_·3H_2_O, and 0.1% Triton X-100, and incubated at 37 °C for one hour. Subsequently, the tissues were decolorized with 95% ethanol.

#### 2.3.2. Root Growth Assay

To investigate the interaction between the isolated bacterial strain and plant roots, four-day-old seedlings of *A. thaliana* ecotype Columbia were transferred to 1/2 MS medium and co-cultured with bacterial inoculants at 23 °C under a 16 h light condition. After six days of co-cultivation, the number of lateral roots and root hairs was recorded.

#### 2.3.3. Analysis of Plant Growth Promotion Effect

Two-week-old *Arabidopsis* seedlings, grown at 23 °C under a 16 h light condition, were treated with the isolated bacterial strain at a density of 1 × 10^8^ CFU/mL once a week for three consecutive weeks. The bacterial isolate was prepared following the procedure described by Tsai et al. (2023) [32]. The fresh weight of the plants was then recorded. A fresh weight of 0.1 g of leaf tissue was extracted in 1 mL of 99% ethanol for 30 min to quantify chlorophyll content, as described by Lichtenthaler (1987) [37]. Following this, supernatants were collected and the absorbance was measured at wavelengths of 664 nm and 648 nm. The chlorophyll content (mg/mL) was calculated using the formula 5.24 × OD_664_ + 22.24 × OD_648_. The procedure described by Yemm and Willis (1954) [38] was adopted to assay the total sugar content in Arabidopsis seedlings after a three-week treatment. A fresh weight of 0.1 g of leaf tissue was ground in 1 mL of 80% ethanol. The extract was dried, resuspended in water, and used to react with 0.4% anthrone solution prepared in concentrated sulfuric acid. The reaction mixture was placed in boiling water for 10 min. The absorbance at a wavelength of 600 nm was measured, and the total sugar content (μg/g) was calculated using a standard curve. To detect starch accumulation, the procedure described by Tsai et al. (2009) [39] was performed. *Arabidopsis* seedlings were dechlorophyllized by boiling in 95% ethanol for 3 min, followed by overlaying 100 µL of Lugol’s solution onto the seedlings and waiting until the leaf samples turned blue-black. Starch content was quantified by boiling *Arabidopsis* seedlings in 80% ethanol, followed by mixing 900 µL of the boiled solution with 100 µL of Lugol’s solution. The absorbance of the mixture was measured at 620 nm, and the starch content (mg/mL) was calculated using a standard curve.

#### 2.3.4. Antioxidant Enzyme Activity, Glucosinolates and Total Phenolic Compounds (TPC)

*Arabidopsis* seedlings were ground in an extraction buffer containing 50 mM potassium phosphate, pH 7, 0.2 mM EDTA, and 1% polyvinylpyrrolidone (PVP). The supernatant was collected for the analysis of antioxidant enzymes, including ascorbate peroxidase (APX) and guaiacol peroxidase (POD), following the procedures described by Chang et al. (2023) [34]. Secondary metabolites, including glucosinolates and TPC, were also analyzed. To measure glucosinolate content, *Arabidopsis* leaf tissues were extracted with 80% methanol, and the glucosinolate content was determined following the procedures described by Mawlong et al. (2017) [40]. To quantify the TPC content, the acetone extract of leaf tissues was added to Folin–Ciocalteu reagent, and the TPC content was determined following the procedures described by Deng et al. (2013) [41]. The TPC content was calculated using a standard curve prepared with known concentrations of gallic acid, and the results were reported in milligrams of gallic acid equivalents (GAE) per gram of extract. All analyses were performed in triplicate.

#### 2.3.5. Western Blot Analysis

Tissues of three-week-old seedlings treated with bacterial culture were collected for protein extraction using methods described by Wang et al. (2003) [42]. Here, 15 μg of total protein was resolved on 10% SDS-PAGE gels and polyclonal antibodies against APX, glutathione peroxidase (GPX), dehydroascorbate reductase 1 (DHAR1), FtsZ1/2, protochlorophilide oxidoreductase (POR), cytosolic fructose-1,6-bisphosphatase (cFBPase), and fructose-1,6 bisphosphate aldolase (ALD) were used for hybridization. All antibodies were obtained from Agrisera (Vännäs, Sweden). Hybridization images were captured using a Chemi-Smart 5000 (Vilber Lourmat, Mlv, France).

### 2.4. Analysis of Abiotic Stress Response

#### 2.4.1. Drought Stress Tolerance

Two-week-old *Arabidopsis* seedlings grown at 23 °C under 16 h light conditions were treated with an isolated bacterial strain at a concentration of 1 × 10^8^ CFU/mL. After treatment, the seedlings were subjected to five days of water deprivation, and cellular oxidative stress was evaluated by measuring H_2_O_2_ levels and the accumulation of malondialdehyde (MDA), a product of lipid peroxidation. To analyze H_2_O_2_ content, 0.1 g of leaf tissue was ground in 1 mL of 80% ethanol, and the resulting supernatant was collected to quantify H_2_O_2_ using the ferrous ion oxidation xylenol orange (FOX) method described by Delong et al. (2002) [43]. The MDA content was quantified by grinding 0.1 g of leaf tissue in 1 mL of 0.25% thiobarbituric acid (TBA) dissolved in 10% trichloroacetic acid (TCA). The mixture was heated at 95 °C for 15 min, and the absorbance of the supernatant was measured at 532 nm and 600 nm. MDA content was determined using the equation provided by Heath and Packer (1968) [44]. After seven days of dehydration, the wilted seedlings were scored. Water was then reintroduced to the dehydrated seedlings, and their fresh weights were measured seven days later.

#### 2.4.2. Heat Stress Tolerance

To assess heat stress tolerance, two-week-old *Arabidopsis* seedlings treated with the isolated bacterial strain were subjected to 45 °C for 10 min. The heat-stressed seedlings were then analyzed for H_2_O_2_ and MDA levels as described above. To examine phenotypic responses to heat stress tolerance, Arabidopsis seedlings treated with the isolated bacterial strain underwent exposure to 45 °C for 20 min, after which they were shifted to a growth temperature of 23 °C. Seedling wilting was evaluated 24 h post-treatment, and fresh weights were measured following a seven-day recovery period at 23 °C.

#### 2.4.3. Salt Stress Tolerance

To assess salt stress tolerance, two-week-old *Arabidopsis* seedlings treated with the bacterial culture were irrigated with 200 mM NaCl every two days for a total of three treatments. After completing the salt stress treatment, the fresh weights of the seedlings were measured. Analyses of chlorophyll, starch, H_2_O_2_, and MDA levels were conducted following the methods described above.

### 2.5. qPCR Analysis

Leaf tissues of three-week-old *Arabidopsis* seedlings treated with bacterial culture were used to extract total RNA following the method described Parcy et al. (1994) [45]. Here, 1 μg of total RNA was used to synthesize cDNA using ImProm-II™ reverse transcriptase (Promega, Madison, WI, USA). The resulting cDNA was used to perform qPCR analysis using SYBR Green Master Mix in the StepOneTM Real-Time PCR System (Thermo Fisher). Primer sequences targeting gene-specific regions were designed based on specific criteria, including a G/C content of 40–60% and a melting temperature range of 58–60 °C, as reported by Chester and Marshak (1993) [46]. The primer sequences used in qPCR amplification are listed in Supplementary Table S1. The qPCR amplification conditions started with an initial denaturation at 95 °C for 10 min, followed by 40 cycles consisting of denaturation at 95 °C for 15 s, annealing at 60 °C for 20 s, and extension at 72 °C for 32 s. Relative gene expression levels were calculated using the 2^−∆∆^Ct method, with *actin 2* (*ACT2*) serving as the reference gene for normalization.

### 2.6. Statistical Analysis

Treatment means were analyzed with SAS statistical software (version 3.8) using ANOVA and Tukey’s test. Differences were considered statistically significant at a *p*-value below 0.05. The results are presented as the mean ± SD based on three replicates.

## 3. Results and Discussion

### 3.1. Characterization of Strain S14

**Figure 1:**
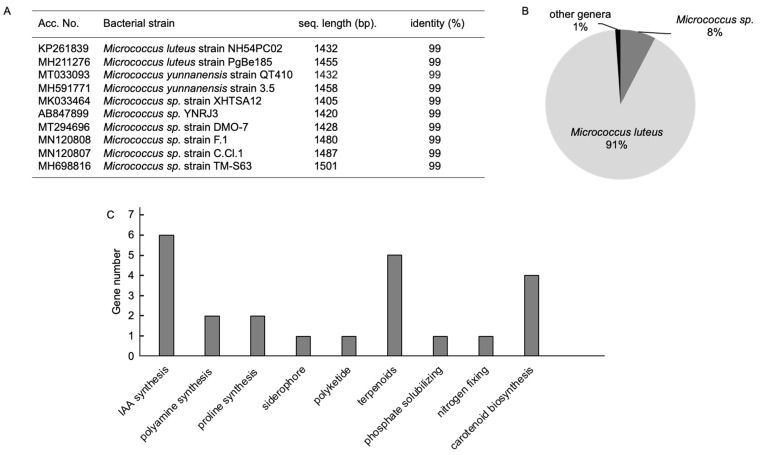
Identification of MlS14. (**A**) The BLAST search results of the 16S rDNA sequences of *Micrococcus* sp. (**B**) The BLASTN results of the genome sequence. (**C**) Gene groups identified in the MlS14 genome associated with plant growth-promoting traits.

The 16S rDNA sequence amplified from the genomic DNA of strain 14 showed 99% sequence identity to bacterial strains of the *Micrococcus* genus (Figure 1A). The de novo assembly of the whole genome of strain S14 revealed 2180 open reading frames (ORFs) that were identified, showing 91.24% sequence similarity to a strain of the *M. luteus* species (Figure 1B). Therefore, this bacterial strain was designated as MlS14. The genome size of MlS14 is approximately 2.4 Mb, with a GC content of 73.1%, similar to that of other bacterial species in this genus [47]. Genes responsible for synthesizing nine categories of metabolites that potentially regulate plant growth were discovered in the MlS14 genome. Of these, the three largest groups of gene clusters were those responsible for the synthesis of IAA, terpenoids, and carotenoids (Figure 1C). Genes associated with plant growth-promoting traits (PGPTs) identified in the MlS14 genome are listed in Table 1.

The MlS14 genome contains five genes from the *trp* operon involved in the synthesis of tryptophan, a precursor for IAA synthesis via tryptophan-dependent pathways [48]. Genes associated with terpenoid synthesis in the MlS14 genome include *CMK*, *ERG9*, *ispA*, *ispDF*, and *ispG* [49,50,51]. MlS14 also harbors multiple genes, including *crtI*, *crtYg*, and *crtYh*, which are involved in the biosynthesis of the C50 carotenoid sarcinaxanthin, which accumulates in *M. luteus* [52]. Additionally, MlS14 contains the *ubiA* gene, encoding a prenyltransferase that participates in C50 carotenoid biosynthesis [53]. Genes identified in the MlS14 genome show potential to produce metabolites including IAA, terpenoids and carotenoids. Microbial IAA is a natural form of auxin. IAA-producing rhizobacteria enhance plant growth by influencing root development, potentially improving nutrient acquisition efficiency and boosting plant growth vigor [48]. Terpenoids and carotenoids exhibit both antioxidant and antimicrobial bioactivity [54,55].

A coding sequence in the MlS14 genome gene was identified to be *phoD*, an alkaline phosphatase that plays a significant role in phosphate solubilizing activity in various PGPRs [56]. The *nif3* gene [57], associated with nitrogen fixation, was also discovered in the MlS14 genome. The microbial activity of phosphate solubilizing and nitrogen fixation can increase soluble phosphate and nitrogen availability for plant nutrient supply [58]. Moreover, gene sequences such as *speE*, involved in the biosynthesis of the polyamine spermidine, and *proC* and *proDH*, responsible for proline synthesis, were identified in the MlS14 genome. Spermidine is a polyamine that plays several essential roles in microorganisms, including promoting biofilm formation, which is vital for microbial survival [59]. Furthermore, spermidine acts as a free radical scavenger, helping to reduce oxidative damage [60]. Research indicates that beneficial rhizobacteria can boost plant growth under salt stress conditions by producing spermidine [61]. Proline is an amino acid that serves as an osmoprotectant, accumulating in many bacterial and plant cells in response to osmotic stress [62]. The genomic sequence of MlS14 revealed the presence of *entC*, a gene essential for the production of the siderophore enterochelin, along with a gene encoding polyketide cyclase, which plays a role in the synthesis of polyketides [63]. Enterochelin demonstrates iron-scavenging activity and is employed as a biocontrol agent [64]. The primary function of microbial polyketides is to serve as antimicrobial agents [65].

### 3.2. Analysis of Growth-Promoting Traits of MlS14

**Figure 2:**
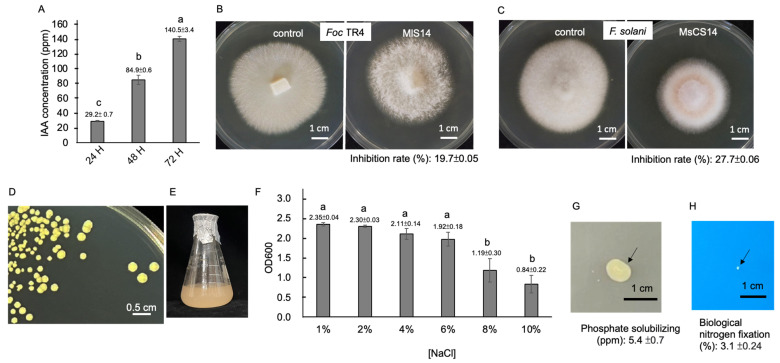
Characterization of MlS14. (**A**) IAA production after 24, 48, and 72 h of culture. Antifungal activity of VOCs produced by MlS14 suppressing mycelial growth of *Fusarium oxysporum* f. sp. *cubense* tropical race 4 (*Foc* TR4) (**B**) and *F. solani* (**C**). Yellow pigment accumulated in the bacterial colonies (**D**) and culture (**E**). MlS14 culture under various NaCl concentrations (**F**). Phosphate-solubilizing activity in PVK medium (arrow indicating clear zone area) (**G**). Detection of nitrogen fixation activity in Jensen’s medium (arrow indicating colony location) (**H**). In each histogram, different letters indicate statistical significance at *p* = 0.05.

The genome of MlS14 revealed multiple ORFs involved in the synthesis of IAA (Table 1). When evaluated for IAA production in the presence of 2 mg/mL tryptophan, MlS14 exhibited a steady increase in IAA levels over time. Notably, MlS14 produced approximately 29 ppm, 85 ppm, and 141 ppm of IAA on the first, second, and third days of culture, respectively (Figure 2A). MlS14 produced 29–141 ppm of IAA over a three-day culture, categorizing it as a high IAA producer, since bacteria that can produce up to 200 ppm of IAA are considered part of this group [66]. These results support the characterization of the *Micrococcus* genus as a high IAA producer [67]. Five genes linked to the synthesis of terpenoids were identified in the MlS14 genome (Table 1). Terpenoids are notable VOCs produced by microorganisms. [68]. In addition to their antioxidant activity, various terpenoid metabolites have been observed to exhibit significant antimicrobial activity [10]. In this study, MlS14-produced volatile compounds showed antifungal activity, suppressing the mycelial growth of *Foc* TR4 and *F. solani*, the pathogens responsible for banana *Fusarium* wilt and *Phalaenopsis* orchid yellow leaf disease, respectively (Figure 2B,C). Four genes responsible for encoding proteins involved in carotenoid metabolite synthesis were identified (Table 1). As a result, MlS14 consistently displayed a yellow colony morphology when cultured on nutrient agar and caused the nutrient broth to turn orange after 72 h of incubation (Figure 2D,E). The carotenoid biosynthesis pathway was identified in *M. luteus* [52]. Carotenoids produced in *Micrococcus* sp. exhibit antioxidant and antibacterial activity [11]. Bacteria are capable of synthesizing a range of osmoprotectants, including glycine betaine, trehalose, and proline, to safeguard themselves against osmotic stress [69]. Two genes in the genome of MlS14 were identified as being involved in the synthesis of proline (Table 1). To evaluate the salt stress tolerance of MlS14, the bacterial culture was grown in media supplemented with varying NaCl concentrations ranging from 1% to 10%. As shown in Figure 2F, the growth of MlS14 was significantly reduced in the presence of 8% NaCl. The MlS14 genome was found to contain the *phoD* and *nif3* gene copies, which are associated with phosphate solubilizing and nitrogen fixation activities, respectively [56,57]. However, our results demonstrate that MlS14 exhibited low activity levels in both phosphate solubilization and nitrogen fixation. When grown on PVK medium, a small clear zone was observed around the MlS14 colony after 14 days of cultivation, and the quantified phosphate-solubilizing activity was approximately 5 ppm after three days of incubation (Figure 2G). MlS14 grew slowly and showed approximately 3% BNF (Figure 2H). These findings suggest that the primary bioactive metabolites produced by MlS14 that may promote plant growth are IAA, terpenoids, and carotenoids.

### 3.3. Root Architecture Affected by MlS14

**Figure 3:**
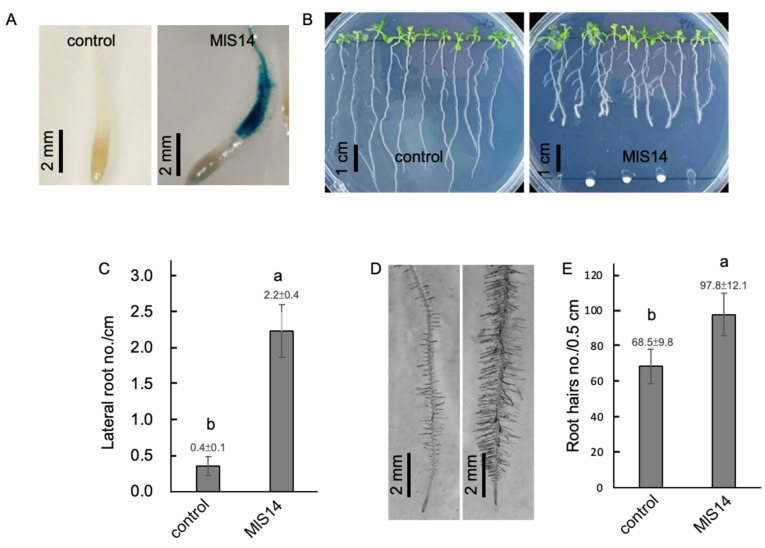
Root development affected by MlS14. (**A**) Histochemical localization of GUS reporter activity in the roots tissues. Six days after co-culture with MlS14, *Arabidopsis* seedlings exhibited changes in root architecture (**B**), lateral root number (**C**), root hair morphology (**D**), and root hair density (**E**). In each histogram, different letters indicate statistical significance at *p* = 0.05.

MlS14 produced a significant amount of IAA (approximately 141 ppm) after three-day culture (Figure 2A). As shown in Figure 3A, the treatment of MlS14 activated GUS expression in a transgenic plant containing the *BA3*::GUS promoter construct, an auxin-inducible promoter [70]. IAA, the predominant naturally occurring auxin in plant cells, is essential for regulating lateral root development [71]. However, high concentrations of exogenous auxin can inhibit primary root elongation [72]. Co-culturing *Arabidopsis* seedlings with MlS14 resulted in changes in root architecture, including shortened primary root growth and increased lateral root growth (Figure 3B). Moreover, the number of lateral roots in the seedlings co-cultured with MlS14 was significantly higher than in the control seedlings (Figure 3C). In the MlS14-treated seedlings, both the density and length of root hairs increased (Figure 3D,E). Comparable changes in root architecture were observed in *Arabidopsis* seedlings inoculated with a different *M. luteus* strain. Moreover, these root phenotypic modifications depend on auxin signaling components [4]. Consistently, MlS14 was able to activate the expression of GUS driven by a promoter containing auxin-responsive elements, indicating the activation of auxin signaling in response to MlS14 treatment. Our results suggest that MlS14 is a high IAA producer, a potent promoter of root development, and a strong activator of the auxin signaling pathway in *Arabidopsis* seedlings.

### 3.4. MlS14 Promoted Growth and Altered Metabolism in Arabidopsis

**Figure 4:**
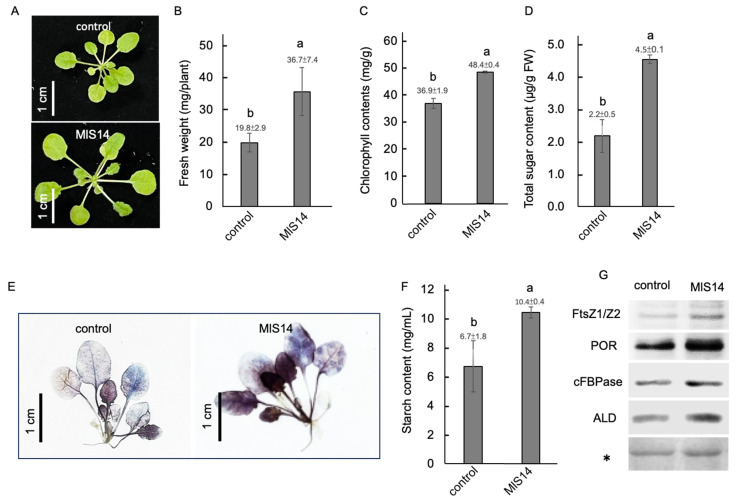
MlS14 promoting *Arabidopsis* growth and metabolism. (**A**) Treatment of MlS14 increased plant size (**A**) and fresh weight (**B**). The leaf tissues were used to analyze the chlorophyll content (**C**) and total sugar content (**D**). Lugol’s iodine solution was used to detect starch–iodine complexes (**E**), and the extracted starch was quantified (**F**). Total proteins isolated from MlS14-treated tissues were used for Western blot analysis with antibodies against proteins, including FtsZ1, protochlorophyllide oxidoreductase (POR), cytosolic fructose-1,6-bisphosphatase (cFBPase), and fructose-1,6-bisphosphate aldolase (ALD). An asterisk indicates Ponceau-stained gels used to verify equal protein loading in each lane (**G**). In each histogram, different letters indicate statistical significance at *p* = 0.05.

In soil-grown *Arabidopsis* seedlings, treatment with MlS14 led to an increase in seedling size and fresh weight (Figure 4A,B). Seedlings treated with MlS14 exhibited significantly higher chlorophyll and total soluble sugar content (Figure 4C,D). Additionally, the MlS14-treated seedlings showed increased blue-black staining due to the starch–iodine reaction (Figure 4E) and accumulated greater amounts of starch in the leaf tissues (Figure 4F). To further confirm the effect of MlS14 on plant growth, the levels of proteins linked to chloroplast function and photosynthesis efficiency were analyzed. As shown in Figure 4G, treatment with MlS14 led to the accumulation of proteins related to photosystem function, including FtsZ1 for chloroplast division [73] and protochlorophyllide oxidoreductase (POR) for chlorophyll biosynthesis [74], as well as cytosolic fructose-1,6-bisphosphatase (cFBPase) and fructose-1,6-bisphosphate aldolase (ALD), both associated with photosynthesis efficiency [75,76]. Exogenous IAA was found to be able to enhance plant photosynthesis rate [77]. Moreover, CO_2_ produced by bacterial respiration in the roots can be transported to the shoot and utilized for photosynthesis. After endophyte colonization, the host plant undergoes changes in its photosynthetic apparatus, resulting in greater photosynthetic efficiency, which in turn stimulate shoot growth and increase plant fresh weight [78]. Our results demonstrate that MlS14 is a potent enhancer of plant photosynthesis, potentially due to increased IAA signaling or elevated CO_2_ levels following its colonization in plant roots.

### 3.5. MlS14 Activated Antioxidant Defense in Arabidopsis

**Figure 5:**
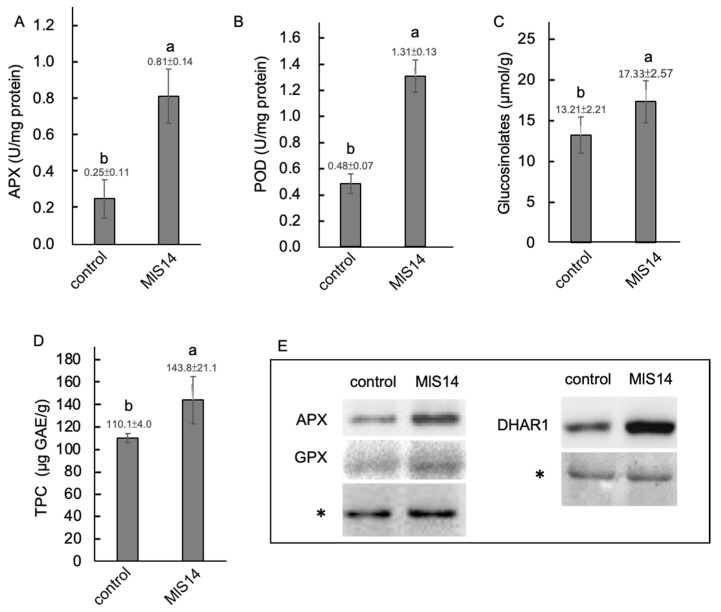
Antioxidant defense system activated by MlS14. Leaf tissues of *Arabidopsis* seedlings treated with MlS14 were harvested for analyses of antioxidant enzymes’ activity including APX (**A**) and POD (**B**), as well as secondary metabolites including glucosinolates (**C**) and total phenolic compounds (TPC) (**D**). Total proteins extracted from Arabidopsis tissues were analyzed by Western blot using antibodies against proteins, including APX, glutathione peroxidase (GPX), and dehydroascorbate reductase 1 (DHAR1). An asterisk indicates Ponceau-stained gels used to verify equal protein loading in each lane (**E**). In each histogram, different letters indicate statistical significance at *p* = 0.05.

Treatment with MlS14 enhanced the antioxidant defense system, including the activation of antioxidant enzymes such as ascorbate peroxidase (APX) and guaiacol peroxidase (POD) (Figure 5A,B), as well as the production of secondary metabolites, including glucosinolates and phenolic compounds (Figure 5C,D). Western blot analyses also revealed the increased accumulation of proteins involved in the ROS scavenging system, including APX, GPX, and DHAR1, in *Arabidopsis* seedlings responding to MlS14 treatment (Figure 5E). APX and DHAR are components of the ascorbate–glutathione cycle [79]. In plants, GPX activity is often linked to glutathione transferase (GST) activity, playing a role in detoxifying toxic molecules generated under stress conditions [80]. In addition to antioxidant enzymes, MlS14 induced the accumulation of glucosinolates and TPC, both of which possess antioxidant activity [81]. Research has shown that bacterial strains from the *Micrococcus* genus can stimulate plant antioxidant enzymes [82,83]. In contrast, our findings suggest that MlS14 treatment effectively activates the antioxidant defense system in *Arabidopsis* seedlings, including the ascorbate–glutathione cycle and secondary metabolites with ROS scavenging properties.

### 3.6. MlS14 Alleviating Drought Stress Impact in Arabidopsis

**Figure 6:**
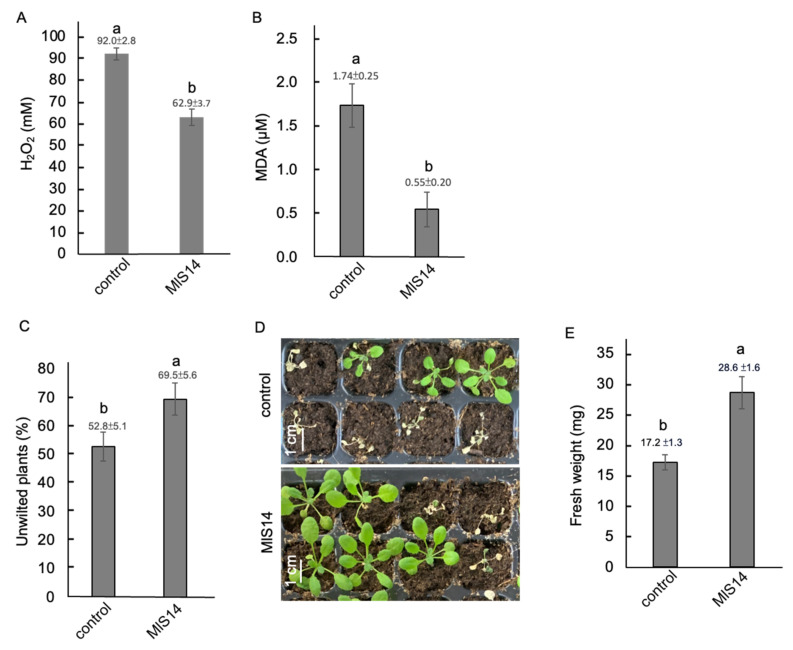
Drought stress tolerance induced by MlS14. Two-week-old *Arabidopsis* seedlings treated with MlS14 were subjected to water depletion for five days. The H_2_O_2_ (**A**) and malondialdehyde (MDA) contents (**B**) were analyzed. Seven days after drought stress, unwilted seedlings were recorded (**C**). Seedlings treated with MlS14 exhibited larger plant size (**D**) and greater fresh weight (**E**) seven days after regaining water supply. In each histogram, different letters indicate statistical significance at *p* = 0.05.

To evaluate the protective effect of MlS14 on *Arabidopsis* seedlings under drought stress, two-week-old seedlings treated with MlS14 were subjected to water-restricted conditions. Five days after the cessation of water supply, MlS14-treated seedlings showed reduced levels of H_2_O_2_ and MDA production compared to the control group (Figure 6A,B). After seven days of water-restricted conditions, a higher percentage of MlS14-treated seedlings remained unwilted, with approximately 69% of the treated plants remaining unwilted compared to around 52% in the control group (Figure 6C). Seven days after rewatering, the MlS14-treated seedlings showed a larger plant size compared to the control seedlings (Figure 6D). Moreover, the fresh weight of the MlS14-treated seedlings was approximately 66% higher than that of the control group (Figure 6E).

### 3.7. MlS14 Increased Heat Stress Tolerance in Arabidopsis

**Figure 7:**
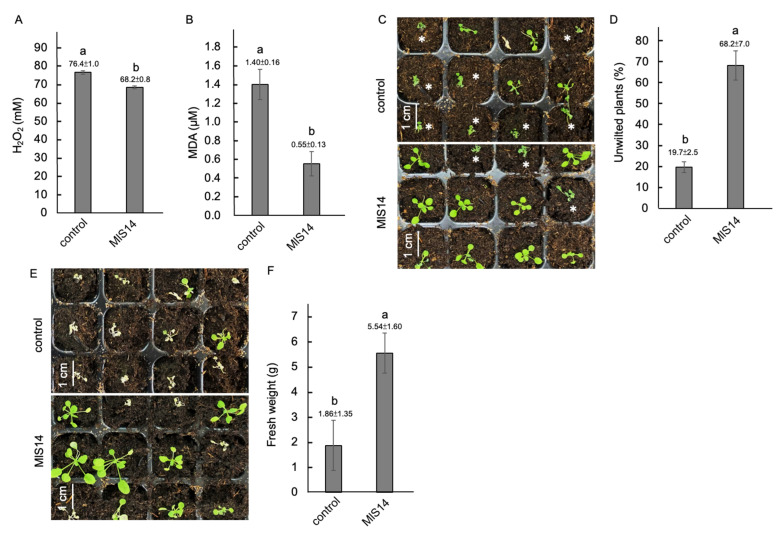
Heat stress tolerance induced by MlS14. Two-week-old *Arabidopsis* seedlings treated with MlS14 were incubated at 45 °C for 10 min and assessed for H_2_O_2_ content (**A**) and MDA content (**B**). Twenty-four hours after returning to 23 °C, the seedlings marked with an asterisk were the wilted ones (**C**). The percentage of unwilted seedlings was then calculated (**D**). MlS14-treated seedlings showed larger plant size seven days after heat stress (**E**) and gained more fresh weight (**F**). In each histogram, different letters indicate statistical significance at *p* = 0.05.

To investigate heat stress tolerance in *Arabidopsis* seedlings treated with MlS14, the seedlings were pretreated with a bacterial inoculant and then exposed to 45 °C for 10 min. During the post-heat stress period, MlS14-treated seedlings accumulated lower levels of H_2_O_2_ and MDA (Figure 7A,B). Twenty-four hours after returning to a normal growth temperature of 23 °C, a higher percentage of unwilted seedlings was observed in the MlS14-pretreated group (Figure 7C), with approximately 68% of the MlS14-treated seedlings remaining unwilted, compared to 20% in the control group (Figure 7D). Additionally, seven days after returning to the normal growth temperature, the MlS14-treated seedlings exhibited significantly larger plant size compared to the control group (Figure 7E), with approximately three times the fresh weight of the control seedlings (Figure 7F).

### 3.8. MlS14 Attenuating Salt Stress Damage in Arabidopsis

**Figure 8:**
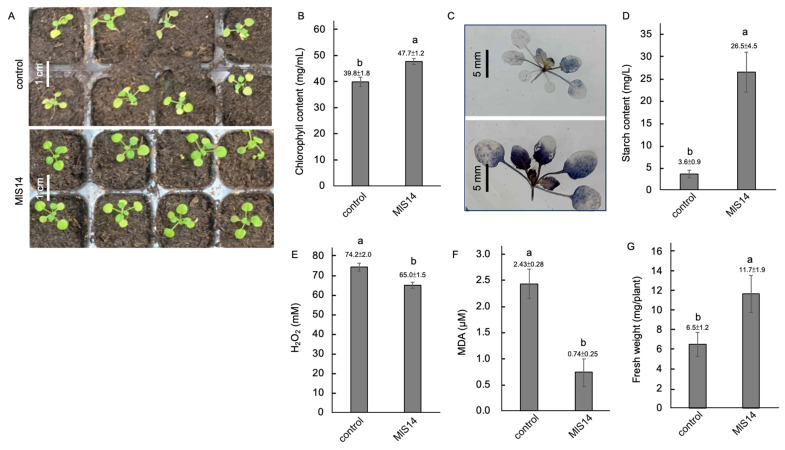
Salt stress tolerance induced by MlS14. Two-week-old *Arabidopsis* seedlings treated with MlS14 were exposed to 200 mM NaCl. Seedling discoloration became evident at the end of the salt stress treatment (**A**), and the chlorophyll contents were measured (**B**). Starch accumulation was detected using lugol’s iodine solution (**C**) and the extracted starch was quantified (**D**). Seedlings were also analyzed for H_2_O_2_ content (**E**), MDA content (**F**), and fresh weight (**G**). In each histogram, different letters indicate statistical significance at *p* = 0.05.

Under treatment with 200 mM NaCl, *Arabidopsis* seedlings pretreated with MlS14 were exposed to salt stress by treatment with 200 mM NaCl. After salt stress treatment, the MlS14-treated seedlings remained green, while the control seedlings showed a yellowish appearance (Figure 8A). Moreover, the MlS14-treated seedlings accumulated higher chlorophyll contents (Figure 8B). Following salt stress, the MlS14-treated seedlings accumulated greater amounts of starch, as evidenced by deeper blue-black staining (Figure 8C). Likewise, the leaf tissues of treated seedlings had a higher starch content compared to the control seedlings (Figure 8D). MlS14 alleviated salt stress damage in *Arabidopsis* by reducing H_2_O_2_ and MDA levels (Figure 8E,F). Moreover, the fresh weight of the treated seedlings was greater than that of the control seedlings (Figure 8G).

Our results show that MlS14 treatment was effective in reducing damage caused by multiple abiotic stress factors, including drought, heat, and salt stress. Various abiotic stresses lead to the accumulation of oxidative stress, which negatively impacts plant physiological functions [84]. Our findings suggest that MlS14 effectively enhances the plant antioxidant defense system, and its biostimulant properties, which are tied to its ability to alleviate oxidative stress, may play a crucial role in improving plant tolerance to abiotic stress. Similarly, previous studies have shown that *Micrococcus* strains alleviate heavy metal stress damage by activating antioxidant enzymes in tomato and rice plants [82,83].

### 3.9. MlS14 Affected Gene Expression in Arabidopsis

**Figure 9:**
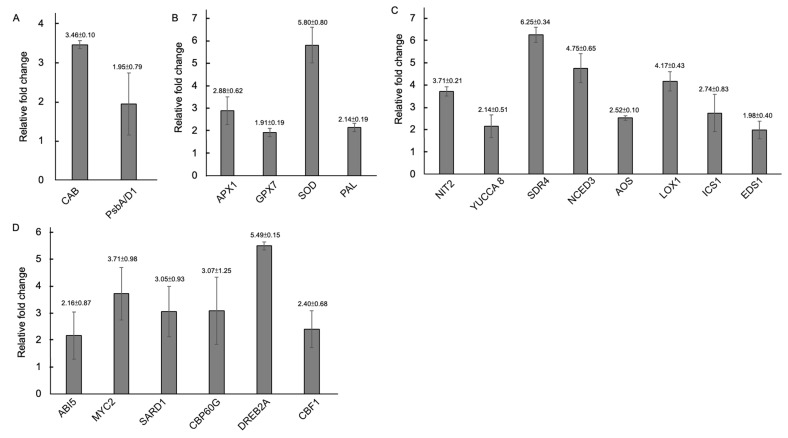
qPCR analysis for gene expression activated by MlS14 treatment. Total RNA isolated from the control and MlS14-treated tissues was used for qPCR analysis using primers specific to genes including *Chlorophyll A/B binding protein 1* (*CAB1*) and *PsbA/D1* (**A**). *Ascorbate peroxidase 1* (*APX1*), *glutathione peroxidase 7* (*GPX7*), *superoxide dismutase 1* (*SOD1*) and *phenylalanine ammonia-lyase 1* (*PAL1*) (**B**). *Nitrilase 2* (*NIT2*), *YUCCA 8*, *short-chain dehydrogenase 4* (*SDR4*), *nine-cis-epoxycarotenoid dioxygenase 3 (NCED3)*, *Lipoxygenase 1* (*LOX1*), *allene oxide synthase* (*AOS*), *isochorismate synthase 1* (*ICS1*) and *enhanced disease susceptibility 1* (*EDS1*) (**C**). *Abscisic acid insensitive 5* (*ABI5*), *MYC2*, *systemic acquired resistance deficient 1* (*SARD1*), *calmodulin-binding protein 60g* (*CBP60g*), *dehydration-responsive element-binding protein 2A* (*DREB2A*) and *C-Repeat Binding Factor 1* (*CBF1*) (**D**).

Our findings reveal that MlS14 had multifaceted effects on *Arabidopsis* growth. To gain insights into the molecular mechanisms underlying the plant growth-promoting effect mediated by MlS14, gene expression linked to various cellular pathways was investigated in *Arabidopsis* seedlings responding to MlS14 treatment. The results of qPCR analysis show that the expressions of *Chlorophyll A/B binding protein 1* (*CAB1*) and *PsbA/D1* encoding photosystem II reaction center protein A were induced to higher levels (Figure 9A); the functions of these two genes are associated with photosynthesis efficiency. Additionally, as shown in Figure 9B, elevated transcript levels were detected for genes encoding antioxidant enzymes such as ascorbate peroxidase 1 (APX1), glutathione peroxidase 7 (GPX7), and superoxide dismutase 1 (SOD1), as well as for phenylalanine ammonia-lyase 1 (PAL1), which plays a role in the synthesis of secondary metabolites with antioxidant properties [85]. The qPCR results for detecting gene expression related to photosynthesis and antioxidant defense are consistent with the Western blot results shown in Figure 4G and Figure 5E. Figure 9C shows that the MlS14 treatment strongly increased the gene expression of *short-chain dehydrogenase 4* (*SDR4*) and *nine-cis-epoxycarotenoid dioxygenase 3* (*NCED3*) involved in ABA biosynthesis [86,87]. Furthermore, increased expression was observed for genes involved in auxin biosynthesis, including *nitrilase 2* (*NIT2*) and *YUCCA 8* [88,89]. Moreover, genes involved in JA synthesis, such as *lipoxygenase 1* (*LOX1*) and *allene oxide synthase* (*AOS*) [90], as well as those involved in SA accumulation, such as *isochorismate synthase 1* (*ICS1*) and *enhanced disease susceptibility 1* (*EDS1*) [91,92], also exhibited higher expression levels. In Figure 9D, MlS14 treatment triggered the expression of several transcription factors involved in the regulation of cellular pathways associated with stress response. These genes include *abscisic acid insensitive 5* (*ABI5*), *MYC2*, *systemic acquired resistance deficient 1* (*SARD1*), *calmodulin-binding protein 60g* (*CBP60g*), *dehydration-responsive element-binding protein 2A* (*DREB2A*), and *C-Repeat Binding Factor 1* (*CBF1*). *ABI5* encodes a basic leucine zipper (bZIP) transcription factor involved in the ABA signaling pathway. Beyond its essential role in controlling seed germination, *ABI5* is crucial for regulating abiotic stress tolerance [93]. *MYC2,* which codes for a protein containing a basic helix–loop–helix (bHLH), plays a regulatory role in transducing JA signaling [94]. *MYC2* also regulates abiotic stress tolerance through the positive regulation of the ABA signaling pathway [95]. Furthermore, *SARD1* and *CBP60g* are two transcription regulators involved in SA biosynthesis [96]. *DREB2A* and *CBF1* (also known as *DREB1B*) are members of the AP2/ERF family, playing a significant role in the regulation of abiotic stress tolerance [97]. Furthermore, *DREB2A* is a key transcription factor controlling the expression of numerous genes responsive to drought, heat, and salt [98].

The qPCR results provide evidence of increased ABA signaling in MlS14-treated seedlings, including elevated expression levels of the ABA biosynthesis genes *SDR4* and *NCED3*, as well as *ABI5*, a positive regulator of ABA signaling [99]. In plant cells, carotenoids serve as essential precursors for ABA biosynthesis [100]. It has been shown that under salt stress conditions, ABA biosynthesis in root tissue depends on the induction of carotenoid synthesis [101]. Therefore, it is plausible that carotenoids produced by MlS14 stimulate ABA synthesis and signaling in *Arabidopsis*. Research has demonstrated that increased ABA signaling can enhance plant tolerance to abiotic stress by elevating antioxidant activity [19,102]. A study also indicates that ABA is essential for the full expression of genes involved in the photosystem function [103]. Furthermore, *ATHB17,* an ABA-responsive HD-Zip transcription factor, regulates the expression of genes involved in the light reactions of photosynthesis. The overexpression of *AtHB17* results in increased tolerance to abiotic stress [104]. These findings suggest that carotenoids produced by MlS14 could play a key role in promoting growth and improving abiotic stress tolerance by activating ABA signaling in *Arabidopsis* seedlings.

Auxin synthesis, like ABA, also depends on the presence of carotenoids. A study demonstrated that the production of both ABA and auxin was impaired in a carotenoid-deficient rice mutant [105]. Beyond its role in regulating root development, auxin contributes to the plant’s adaptive response to abiotic stress. The activation of *YUCCA6*, a key component of the tryptophan-dependent auxin biosynthesis pathway, may enhance the plant’s resistance to drought stress [106]. Furthermore, reducing free auxin by overexpressing the *GH3* gene, which facilitates the conversion of free auxin to its conjugated form, triggered a hypersensitive response to drought stress and lowered ABA concentration [107]. Further crosstalk between ABA and auxin signaling is demonstrated by the fact that *OsbZIP46*, a transcription factor involved in the ABA signaling pathway, could activate *YUCCA8* expression and regulate root growth in rice [108]. Based on this study, the activated auxin signaling in MlS14-treated plants may be attributed to IAA produced by MlS14 or to increased ABA signaling induced by microbial carotenoids. MlS14 produced a high level of IAA. Exogenous auxin has been reported to regulate photosynthetic efficiency and antioxidant activity to mitigate copper toxicity in *Brassica juncea* [109]. The increased auxin signal might play multiple roles in regulating root growth and stress tolerance in *Arabidopsis*. The expression of genes involved in biosynthesis and the responses of JA and SA signals was up-regulated by MlS14. The JA and SA signaling pathways play a role in induced disease resistance mediated by various beneficial bacteria [110]. Beyond their involvement in defense against biotic stress, JA and SA also help improve plant tolerance to abiotic stress by activating antioxidant activity [23,24]. A study has shown an interaction between ABA and JA signaling, where ABA can affect JA biosynthesis and play a role in the plant defense response [111]. An accumulation of oxidative stress may lead to an increase in SA levels [112]. Therefore, the IAA and carotenoids produced by MLS14 may primarily modulate ABA and auxin to positively influence plant growth and stress tolerance.

## 4. Conclusions

Using de novo whole-genome assembly and biochemical analysis, IAA, terpenoids, and carotenoids were identified as key bioactive metabolites produced by the bacterial strain MlS14, potentially contributing to plant growth promotion. Treatment with MlS14 enhanced photosynthesis, strengthened the antioxidant defense system, and increased *Arabidopsis* tolerance to drought, heat, and salt stress. This treatment also activated gene expression associated with multiple hormone signaling pathways, including ABA, auxin, JA, and SA, in *Arabidopsis* seedlings. Given the observed phenotypic changes and transcriptional alterations, microbial IAA and carotenoids likely play primary roles in promoting plant growth and enhancing stress tolerance in *Arabidopsis* by modulating ABA and auxin signaling pathways.

## Figures and Tables

**Table 1:** The PGPT-associated genes.

Acc. No.	Gene	Acc. No.	Gene
IAA synthesis			
WP_206480477	*trpA*	EFD51043	*trpB*
WP_098471668	*trpC*	WP_206481878	*trpB*
WP_101962912	*trpD*	WP_060774686	*trpE*
Spermidine synthesis			
WP_041103603	*speE*	WP_002855478	*speE*
Proline synthesis			
PFH05939	*prodh*	PZP23918	*proC*
Siderophore synthesis			
PFH06113	*entC*		
Polyketide synthesis			
WP_049159382	*polyketide cyclase*		
Terpenoids synthesis			
WP_192592997	*CMK*	WP_095347529	*ispDF*
WP_049158409	*ispA*	OOL27499	*ispG*
WP_065572632	*ERG9*		
Phosphate solubilizing			
WP_201294449	*phoD*		
Nitrogen fixation			
WP_073115524	*nif3*		
Carotenoid synthesis			
WP_020626540	*ubiA*	PFH06102	*crtI*
WP_049158405	*crtYg*	WP_126860818	*crtYh*

## Data Availability

No new data are created.

## Supplementary Materials

The following supporting information can be downloaded at: https://www.mdpi.com/article/10.3390/microorganisms12112283/s1, Supplementary Table S1: Primer sequences for qPCR analysis.
