# Supplementary material for: Decoding the Impact of a Bacterial Strain of Micrococcus luteus on Arabidopsis Growth and Stress Tolerance
Source: Microorganisms. 2024 Nov 10;12(11):2283. doi: 10.3390/microorganisms12112283 (PMC11596720; doi:10.3390/microorganisms12112283)
Supplement: Supplementary file 1 [file microorganisms-12-02283-s001.zip › microorganisms-3261722-supplementary.pdf]

**Supplementary Materials:**

Supplementary Table S1. Primer sequences for qPCR analysis

| **Gene** | **Acc. No.** | **primer sequence** |
|---|---|---|
| Actin2 | AT3G18780 | forward 5′ CGGTAACATTGTGCTCAGTG3′ reverse 5′GTGAACGATTCCTGGACCTG3′ |
| SARD1 | AT1G73805 | forward 5′ GCACTTATCGATGGTCATGT3′ reverse 5′ CCGCTGGAGTCGTTATATAC3′ |
| CBP60G | AT5G26920 | forward 5′ GCGAGTGCTAGTGGAGGAGA3′ reverse 5′ CTGGCAGTTGTGTGTCTCCG3′ |
| MYC2 | AT1G32640 | forward 5′ GCGAGTGCTAGTGGAGGAGA3′ reverse 5′ GCTCTGAGCTGTTCTTGCGT3′ |
| LOX1 | AT1G55020 | forward 5′ CGGACAGTATCCAGTTGCTG3′ reverse 5′ GTTCTTGAGAGTGTCGTCGT3′ |
| YUCCA8 | AT4G28720 | forward 5′ CGTCTCAAGCTTCACCTTCC3′ reverse 5′ TCGACTCACTCTTCGACACG3′ |
| NCED3 | AT3G14440 | forward 5′CGGTGGTTTACGACAAGAAC3′ reverse 5′ CCATCCCTGCTTCGAGGTTG3′ |
| SDR4 | AT3G29250 | forward 5′ GCTTCTAAGCACGCGCTTCT3′ reverse 5′ TCATGAGCTTAACGACGCTA3′ |
| ABI5 | AT2G36270 | forward 5′ ATGTGAAGGAGGAGAACCTC3′ reverse 5′ GTGGACAACTCGGGTTCCTC3′ |
| CAB1 | AT1G29930 | forward 5′ GGAAGATTGGCTATGTTCTC3′ reverse 5′ CGGGAACAAAGTTGGTGGCG3′ |
| PsbA/D1 | ATCG00020 | forward 5′ CTTCTTAGCGGCTTGGCC3′ reverse 5′ CTCAACAGCAGCTAGGTC3′ |
| SOD | AT1G08830 | forward 5′ CGGTTGCATGTCTACTGG3′ reverse 5′ GCCCTGGAGACCAATGAT3′ |
| PAL1 | AT2G37040 | forward 5′ GTGAGGGAAGAGCTTGGAA3′ reverse 5′ CATATTGGAATGGGAGCTCC3′ |
| EDS1 | AT3G48090 | forward 5′ GAATTCGGGATCCGAGTGCG3′ reverse 5′ CCATCATATAGTCTCGCAGAG3′ |
| DREB2A | AT5G05410 | forward 5′ ATGGCAGTTTATGATCAGAG3′ reverse 5′ GTTCTCCAGATCCAAGTAAC3′ |
| CBF1 | AT4G25490 | forward 5′ GCCCTCCGTGGCCGATCAGC3′ reverse 5′ CCAAAGCGACACGTCACCAT3′ |
